# Low-dose rituximab for MPO-ANCA-associated hypertrophic pachymeningitis with delayed onset of renal involvement: a case report

**DOI:** 10.3389/fmed.2026.1843169

**Published:** 2026-05-21

**Authors:** Haohu Wu, Guangzhi Sun, Tongguan Li, Mengjiao Yao, Maiqi Liu, Yanfeng Hou

**Affiliations:** 1 Jining Medical University, Jining, Shandong, China; 2 Department of Orthopedic Surgery, The First Affiliated Hospital of Shandong First Medical University & Shandong Provincial Qianfoshan Hospital, Jinan, Shandong, China; 3 The First Affiliated Hospital of Shandong First Medical University & Shandong Provincial Qianfoshan Hospital, Jinan, Shandong, China; 4 Department of Rheumatology and Autoimmunology, The First Affiliated Hospital of Shandong First Medical University & Shandong Provincial Qianfoshan Hospital, Shandong Province University Clinical Immunology Translational Medicine Laboratory, Jinan, Shandong, China

**Keywords:** ANCA-associated vasculitis, hypertrophic pachymeningitis, microscopic polyangiitis, MPO-ANCA, rituximab

## Abstract

Hypertrophic pachymeningitis (HP) is a rare disorder characterized by focal or diffuse inflammatory thickening of the dura mater. Patients may present with prolonged localized central nervous system involvement and often lack the typical manifestations of ANCA-associated vasculitis (AAV), making diagnosis and treatment challenging. We report the case of a 55-year-old woman who presented with recurrent headache, right eye pain, and visual decline. Laboratory testing showed positivity for myeloperoxidase anti-neutrophil cytoplasmic antibody (MPO-ANCA), and brain magnetic resonance imaging (MRI) demonstrated extensive dural thickening and enhancement involving the bilateral cerebral convexities, falx cerebri, and cerebellar tentorium. Based on the clinical, laboratory, and imaging findings, she was diagnosed with MPO-ANCA-associated hypertrophic pachymeningitis. During follow-up, the patient experienced multiple relapses and showed poor tolerance to several immunosuppressive agents. She was subsequently treated with low-dose rituximab (100 mg) for induction and maintenance therapy. After treatment, her clinical symptoms improved, and the dural thickening and enhancement on imaging were alleviated. During follow-up, she developed proteinuria and hematuria, and renal biopsy ultimately confirmed microscopic polyangiitis (MPA). After intensified treatment, inflammatory markers and renal involvement were brought under control. This case suggests that, in patients with recurrent MPO-ANCA-associated hypertrophic pachymeningitis who are intolerant to multiple immunosuppressive agents, an individualized low-dose rituximab regimen may help achieve a period of clinical and radiological remission.

## Introduction

Hypertrophic pachymeningitis (HP) is a rare disease characterized by focal or diffuse inflammatory thickening of the dura mater and often presents clinically with persistent headache, cranial neuropathy, and visual or hearing impairment (1, 2). The etiology of HP is complex and may include infection, malignancy, autoimmune disease, and idiopathic causes. In recent years, with advances in immunological research, ANCA-associated hypertrophic pachymeningitis (ANCA-HP) has gradually been recognized as an important immunological subtype of HP, among which myeloperoxidase-ANCA (MPO-ANCA) positivity is the most common, particularly in Asian populations (1, 2). At present, glucocorticoids combined with immunosuppressive agents, such as cyclophosphamide, azathioprine, or methotrexate, are generally regarded as first-line treatment for ANCA-HP (1). However, in some patients, conventional therapy fails to achieve sustained remission, and clinical management is complicated by glucocorticoid dependence, frequent relapse during tapering, or poor tolerance to adverse effects of immunosuppressive agents. Under these circumstances, effective subsequent treatment strategies remain poorly defined, and identifying a safer and more effective option for refractory or relapsing MPO-ANCA-associated HP remains a major clinical challenge.

When ANCA-HP is accompanied by multiorgan involvement, such as renal, pulmonary, or ear-nose-throat manifestations, patients are usually classified as having ANCA-associated vasculitis (AAV) (1). In recent years, B-cell-targeted rituximab has been shown to be effective for both induction and maintenance treatment of AAV and has been recommended as an important therapeutic option in several guidelines (3, 4). However, for patients with ANCA-HP who have not yet progressed to systemic AAV, there is still no well-defined rituximab dosing strategy. Standard-dose rituximab may increase the risk of adverse events, particularly infection, and may also impose an additional financial burden. In this context, low-dose rituximab may represent a potentially valuable therapeutic strategy worthy of further exploration. Here, we report a patient with MPO-ANCA-associated HP who responded poorly to glucocorticoids combined with immunosuppressive therapy but achieved favorable clinical and radiological remission after low-dose rituximab treatment, with the aim of providing clinically relevant evidence for the management of similar refractory cases.

## Case presentation

A 55-year-old woman was admitted because of recurrent headache and right eye pain. The headache was characterized by paroxysmal distending pain in the vertex and bilateral temporal regions, accompanied by nausea, vomiting, persistent right eye pain, conjunctival hyperemia, and tearing. According to the available medical records, the patient had no obvious past medical history or family history. No previous chronic kidney disease, pulmonary disease, malignancy, chronic infection, or previously diagnosed systemic autoimmune disease was documented before the onset of the current illness. Detailed psychosocial history was limited in the available records; however, the prolonged disease course, recurrent headache, ocular pain, visual decline, repeated relapses, and intolerance to multiple immunosuppressive agents imposed a considerable treatment burden.

On physical examination at presentation, the patient was conscious and oriented. Neurological examination showed no characteristic neurological findings, except for decreased visual acuity in the right eye. There was no disturbance of consciousness, meningeal irritation sign, obvious limb weakness, sensory deficit, pathological reflex, or cerebellar ataxia. Ocular examination revealed right eye pain with conjunctival hyperemia and tearing. No obvious skin rash, peripheral edema, pulmonary signs, arthritis, or other clear systemic vasculitic manifestations were observed at the initial presentation.

**Figure 1:**
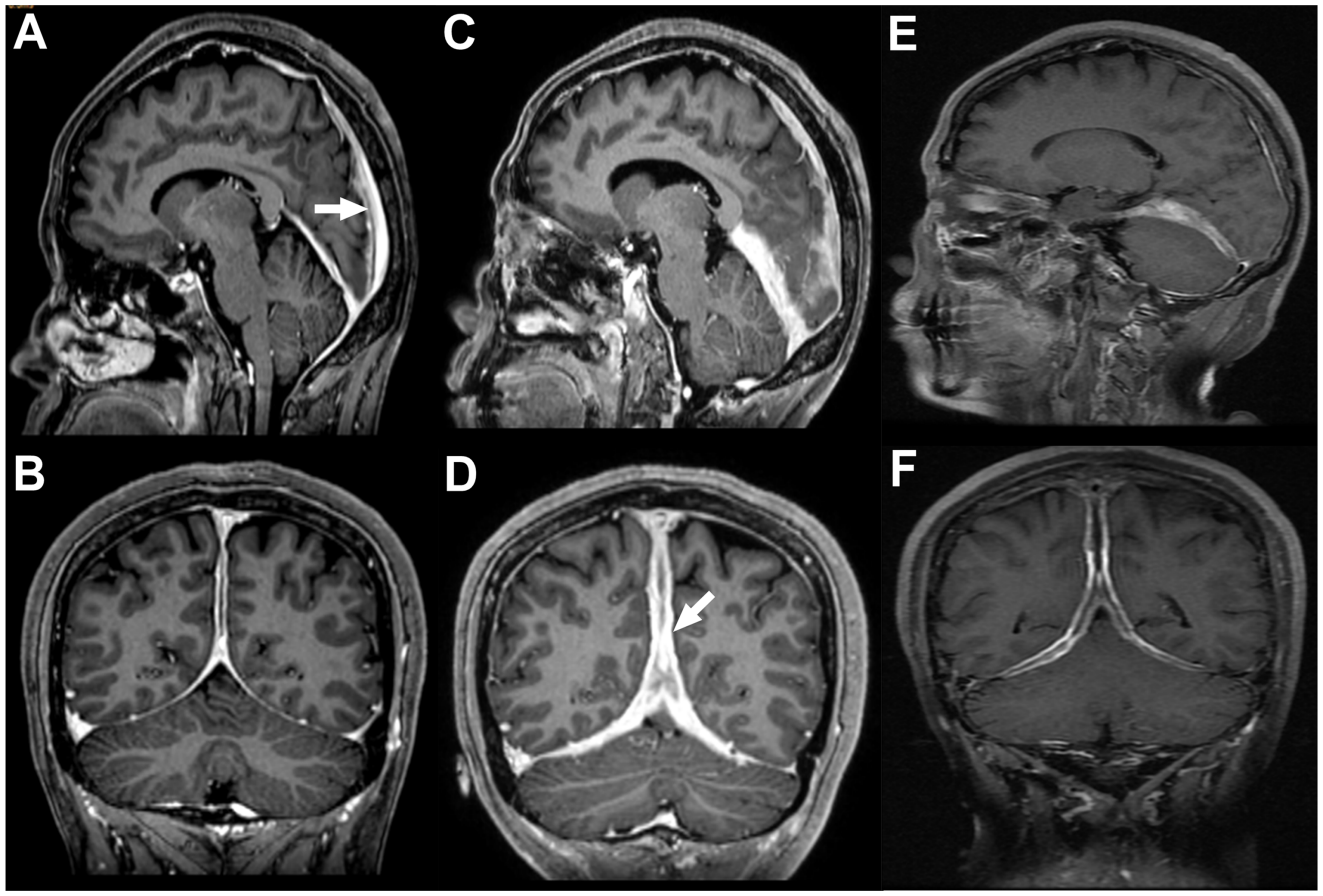
Contrast-enhanced sagittal **(A,C,E)** and coronal **(B,D,F)** T1-weighted images obtained on May 24, 2021 **(A,B)**, April 22, 2022 **(C,D)**, and October 11, 2023 **(E,F)**. White arrows indicate areas of dural thickening and contrast enhancement. Compared with earlier examinations, both the extent and intensity of enhancement were substantially reduced on October 11, 2023.

In early 2019, she presented to the ophthalmology department because of right eye pain. Local pathology suggested right scleritis. Her symptoms improved after three retrobulbar dexamethasone injections, and she was maintained on eye drops and oral prednisone (4.5 mg/day). In August 2019, she was referred to the neurology department because of visual decline. Laboratory testing showed positivity for perinuclear anti-neutrophil cytoplasmic antibody (P-ANCA), and enzyme-linked immunosorbent assay revealed a myeloperoxidase antibody level of 199.04 RU/mL (reference range, 0–20 RU/mL). Complete blood count, liver and renal function, urinalysis, C-reactive protein, and screening for hepatitis B, hepatitis C, syphilis, and HIV were all unremarkable. Antinuclear antibodies (ANA), anti-double-stranded DNA antibodies (anti-dsDNA), anticardiolipin antibodies (aCL), and PR3-ANCA were negative. Serum IgG, IgG4, IgA, IgM, and complement levels were within normal ranges. Chest computed tomography showed only nodular changes. Contrast-enhanced brain MRI demonstrated diffuse thickening and enhancement of the dura involving the bilateral cerebral convexities, falx cerebri, and cerebellar tentorium (Figures 1A,D). Lumbar puncture revealed an intracranial pressure of 140 mmH_2_O, mildly elevated cerebrospinal fluid (CSF) protein, and an increased proportion of mononuclear cells. Tuberculosis polymerase chain reaction (TB-PCR) and oligoclonal bands were negative, and cerebrospinal fluid cytology showed no malignant cells. Repeated microbiological studies of both serum and cerebrospinal fluid were negative.

A structured diagnostic assessment was performed to exclude the main alternative causes of hypertrophic pachymeningitis. First, intracranial hypotension syndrome was considered unlikely because the patient had no typical orthostatic headache, and lumbar puncture did not show low cerebrospinal fluid pressure. Second, infectious pachymeningitis was considered less likely because repeated microbiological studies of both serum and cerebrospinal fluid were negative, including tests for tuberculosis, and screening for hepatitis B, hepatitis C, syphilis, and HIV was unremarkable. Third, malignant pachymeningitis was not supported because cerebrospinal fluid cytology showed no malignant cells, and chest computed tomography did not reveal findings suggestive of an underlying malignancy. Fourth, IgG4-related disease (IgG4-RD) was considered less likely because serum IgG4 was within the normal range and no typical systemic manifestations were identified. Other systemic autoimmune diseases were also considered unlikely because antinuclear antibodies, anti-double-stranded DNA antibodies, anticardiolipin antibodies, and PR3-ANCA were negative, while complement levels were within normal ranges. Because no systemic involvement, such as pulmonary or renal involvement, was identified at that time, the patient did not fulfill the classification criteria for ANCA-associated vasculitis. Based on the clinical manifestations, diffuse dural thickening and enhancement on contrast-enhanced MRI, and MPO-ANCA positivity, MPO-ANCA-associated hypertrophic pachymeningitis was considered (Table 1).

**Table 1:** Structured differential diagnosis of hypertrophic pachymeningitis in this patient.

Diagnostic consideration	Evidence item	Key findings in this patient	Interpretation
Infectious pachymeningitis	Symptoms/signs	Recurrent headache/ocular pain	Superimposed infection possible infection alone insufficient
		No fever at onset; no meningeal irritation signs	
	Past history	No chronic infection documented; HBV/HCV/HIV/syphilis (−)	
	CSF/microbiology	Initial serum/CSF microbiology (−); CSF TB-PCR (−); CrAg/AFB/fungal/bacterial cultures repeatedly (−); later CSF culture/mNGS: *Staphylococcus aureus* (*+*)	
	Limitation	No dural biopsy	Not completely excluded
Malignancy-related involvement	Symptoms/signs	No cancer-related systemic symptoms documented; neuro exam without focal mass-related deficits except decreased right visual acuity	No clear supporting evidence
	Past history	No known malignancy or family history; female tumor marker panel: no malignancy-suggestive abnormality	
	CSF/imaging	CSF cytology: no malignant cells	
		Chest CT: nodular changes only	
	Limitation	No dural biopsy; no whole-body CT or 18F-FDG PET/CT	Less likely, not completely excluded
IgG4-related HP	Symptoms/signs	No lacrimal/salivary gland enlargement, orbital mass/proptosis, pancreatobiliary symptoms, lymphadenopathy, or retroperitoneal/renal involvement documented	No clear supporting evidence
	Serology	Serum IgG and IgG4 within normal ranges	
	Limitation	No dural biopsy	
Other systemic autoimmune disease	Symptoms/signs	No rash, arthritis, edema, pulmonary signs, or other systemic vasculitic manifestations at onset	Alternative systemic autoimmune disease less likely
	Serology	ANA (−), anti-dsDNA (−), aCL (−), RF (−), PR3-ANCA (−); complement normal	
Intracranial hypotension syndrome	Symptoms/signs	Orthostatic headache (−)	Unlikely
	CSF/MRI	CSF pressure not low; elevated CSF pressure during relapse; MRI pattern not typical for intracranial hypotension	
MPO-ANCA-associated HP/AAV	Symptoms/signs	Recurrent headache, right eye pain, right scleritis, decreased right visual acuity	Most plausible clinical-radiological diagnosis
	Serology/CSF/MRI	MPO-ANCA (+), PR3-ANCA (−); inflammatory CSF findings; diffuse dural thickening/enhancement	
	Follow-up/pathology	Renal biopsy: PICGN, fulfilled MPA criteria	
	Limitation	No dural biopsy	Not histopathologically confirmed

PICGN, pauci-immune ANCA glomerulonephritis; AAV, ANCA-associated vasculitis; aCL, anticardiolipin antibody; AFB, acid-fast bacilli; ANA, antinuclear antibody; anti-dsDNA, anti-double-stranded DNA antibody; CrAg, cryptococcal antigen; CSF, cerebrospinal fluid; CT, computed tomography; HBV, hepatitis B virus; HCV, hepatitis C virus; HIV, human immunodeficiency virus; HP, hypertrophic pachymeningitis; IgG, immunoglobulin G; IgG4-RD, immunoglobulin G4-related disease; mNGS, metagenomic next-generation sequencing; MPA, microscopic polyangiitis; MPO-ANCA, myeloperoxidase antineutrophil cytoplasmic antibody; MRI, magnetic resonance imaging; 18F-FDG PET/CT, 18F-fluorodeoxyglucose positron emission tomography/computed tomography; PICGN, pauci-immune crescentic glomerulonephritis; PR3-ANCA, proteinase 3 antineutrophil cytoplasmic antibody; RF, rheumatoid factor; TB-PCR, tuberculosis polymerase chain reaction.

During hospitalization, she was treated with methylprednisolone (40 mg/day) combined with azathioprine (25 mg/day), and her symptoms improved. However, azathioprine was discontinued because of abnormal liver function. She was then maintained on oral prednisone (50 mg/day) with gradual tapering, but her symptoms relapsed when the dose was reduced to approximately 25 mg/day. In January 2020, repeat testing showed that the MPO antibody titer had increased to >400 RU/mL. She received methylprednisolone pulse therapy and intravenous cyclophosphamide pulse therapy (0.6 g), but cyclophosphamide was discontinued because of marked gastrointestinal adverse effects. Intrathecal administration of dexamethasone (10 mg) was subsequently attempted, after which her headache improved rapidly. She was then maintained on prednisone (22.5 mg/day) and methotrexate (12.5 mg/week). In July 2020, she was readmitted because of worsening headache and right eye pain, with persistently positive MPO antibody titers. Following intrathecal administration of dexamethasone (10 mg) combined with methotrexate (10 mg), her symptoms improved again, and weekly intravenous cyclophosphamide (0.2 g) was added. After discharge, she continued prednisone (10 mg/day) and methotrexate (12.5 mg/week), but immunosuppressive therapy was stopped approximately 2 months later because of gastrointestinal intolerance and abnormal liver function. She remained on prednisone (12.5 mg/day) alone.

**Figure 2:**
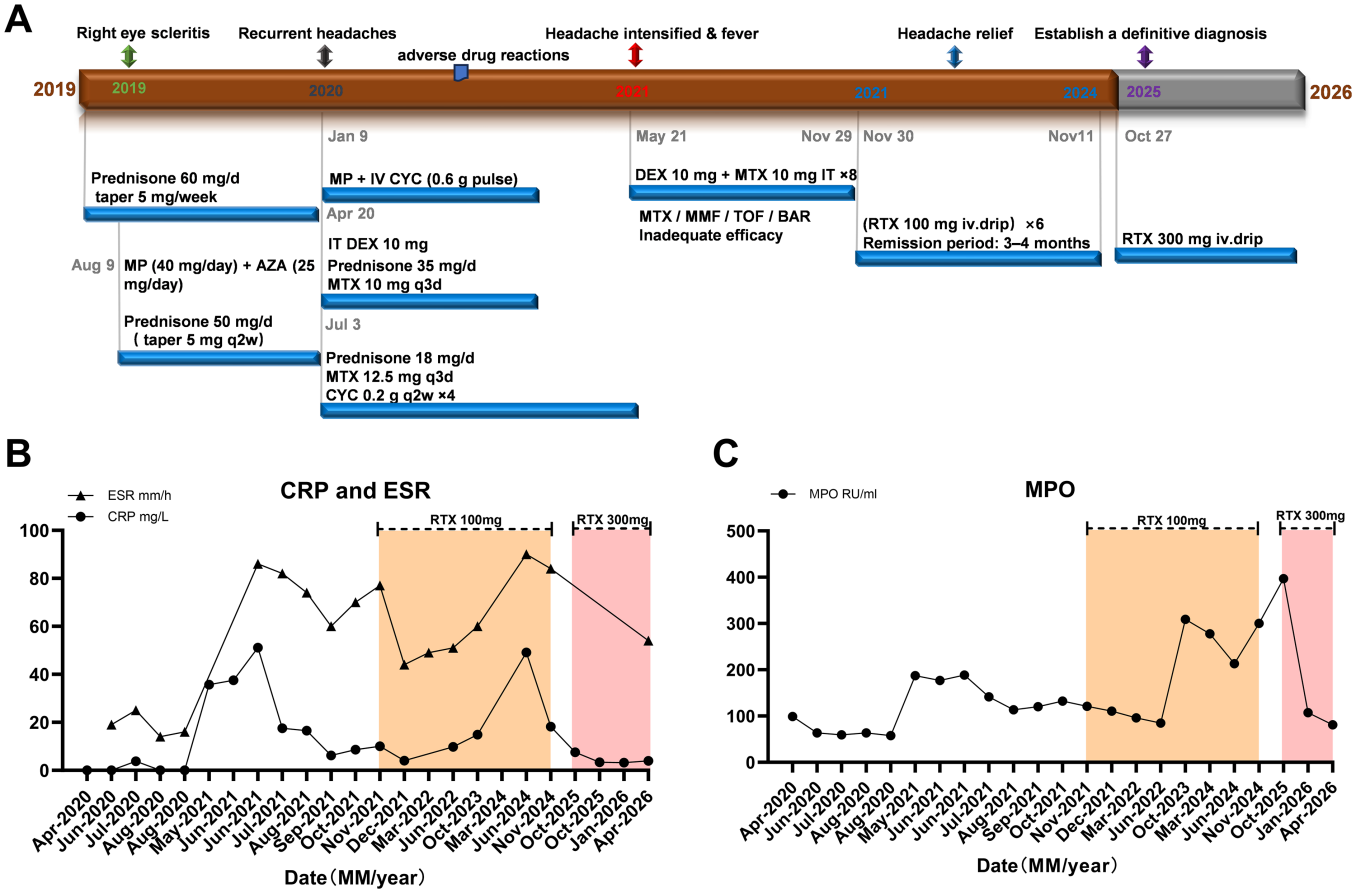
Clinical course and laboratory parameters reflecting disease activity in this patient. **(A)** Timeline of medication use. **(B)** Changes in erythrocyte sedimentation rate (ESR) and C-reactive protein (CRP). **(C)** Changes in serum MPO-ANCA levels throughout the disease course.

In May 2021, she again developed severe headache and right eye pain, accompanied by blurred vision, nausea, and vomiting. On admission, her highest recorded body temperature was 37.5 °C. Lumbar puncture showed elevated intracranial pressure, and the Pandy test in cerebrospinal fluid was positive. Cerebrospinal fluid culture and metagenomic next-generation sequencing (mNGS) suggested *Staphylococcus aureus* infection. She was treated with vancomycin for anti-infective therapy and mannitol for intracranial pressure reduction, after which infection-related indices improved; however, the headache continued to recur. Ongoing inflammatory activity of hypertrophic pachymeningitis was therefore considered the main driving factor, and she received intravenous cyclophosphamide pulse therapy (0.6 g), followed by another intrathecal injection of dexamethasone (10 mg) combined with methotrexate (10 mg). Her symptoms improved only transiently, and the duration of remission gradually shortened. Subsequent maintenance treatment with mycophenolate mofetil, tofacitinib, and baricitinib was attempted, but all were discontinued because of drug intolerance, and the disease exhibited a refractory, relapsing course (Figure 2A).

**Figure 3:**
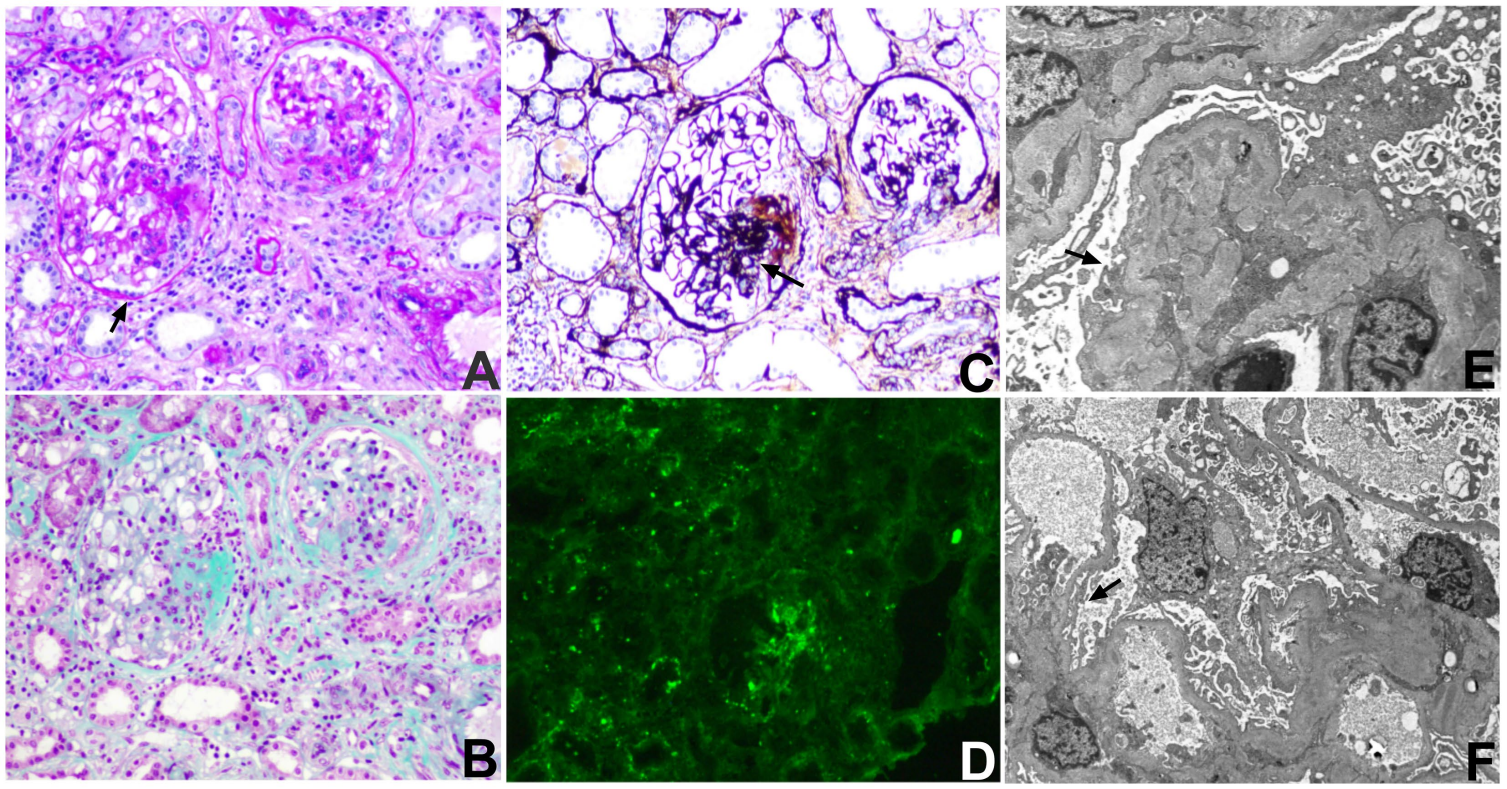
Light microscopy showed crescentic glomerular injury with tubulointerstitial damage and focal fibrosis **(A,B)**. PASM staining showed capillary loop and basement membrane abnormalities **(C)**. Immunofluorescence showed a pauci-immune pattern **(D)**. Electron microscopy showed irregular capillary loops, basement membrane wrinkling, and partial podocyte foot process effacement **(E,F)**. Arrows indicate crescent formation in the glomeruli **(A,B)** and focal podocyte foot process effacement **(F)**.

In November 2021, rituximab therapy was initiated at a dose of 100 mg per infusion. Her clinical symptoms improved markedly, and follow-up contrast-enhanced MRI showed reduced dural thickening and enhancement compared with pretreatment imaging (Figures 1A–F). By 2024, she had received six maintenance infusions, with each remission lasting approximately 4–6 months. In October 2025, she presented with a 3-month history of foamy urine and nocturia. Laboratory testing showed proteinuria, microscopic hematuria, and elevated serum creatinine. Renal biopsy revealed necrotizing crescentic glomerulonephritis, and immunofluorescence showed no immune complex deposition (Figure 3), consistent with pauci-immune crescentic glomerulonephritis. According to the 2022 ACR/EULAR classification criteria, her total score was 9, and she was diagnosed with microscopic polyangiitis (MPA). Intensified treatment with rituximab (300 mg) was then administered. Rituximab (300 mg) was re-administered 6 months later. Follow-up evaluation demonstrated marked reductions in MPO-ANCA, ESR, and CRP levels (Figures 2B,C). Her condition has not worsened further, and she remains under follow-up.

## Discussion

This case describes a rare patient with MPO-ANCA-associated hypertrophic pachymeningitis complicated by microscopic polyangiitis. After excluding autoimmune diseases, infection, systemic disorders, and malignancy as causes of dural thickening, and given the presence of P-ANCA and MPO positivity together with contrast-enhanced brain MRI showing diffuse thickening and enhancement of the falx cerebri and cerebellar tentorium, the diagnosis of MPO-ANCA-associated HP was considered appropriate in the absence of typical systemic organ involvement.

The relationship between MPO-ANCA-associated HP and AAV remains controversial, although the two are closely related. On the one hand, previous studies have suggested that MPO-ANCA-positive HP may represent a central nervous system-limited subtype of AAV (1, 2). On the other hand, some reports have shown that a subset of patients do not develop systemic involvement even after long-term follow-up, raising the possibility that MPO-ANCA-associated HP may also represent a relatively distinct clinical entity (5, 6). Notably, our patient initially had only localized central nervous system involvement, but gradually developed proteinuria, microscopic hematuria, and elevated serum creatinine during follow-up. Renal biopsy subsequently confirmed pauci-immune necrotizing glomerulonephritis, and the patient ultimately fulfilled the classification criteria for MPA. This disease course supports the possibility that MPO-ANCA-associated HP and AAV may represent a continuum rather than two entirely separate disorders. ANCA-positive HP may progress slowly (7), and renal function may remain completely normal at an early stage (8, 9). Therefore, even patients without apparent systemic involvement at presentation require long-term monitoring and dynamic reassessment, particularly with respect to renal manifestations.

De Virgilio et al. (10) noted in their review that there is currently no consensus regarding the treatment of immune-mediated hypertrophic pachymeningitis, whether idiopathic or secondary. In clinical practice, treatment of ANCA-associated HP is largely extrapolated from therapeutic strategies used for AAV, including recent guidelines such as the 2025 British Society for Rheumatology recommendations and the 2024 KDIGO guideline (3, 4). Most patients are initially treated with glucocorticoids and, in cases of relapse, often receive additional immunosuppressive agents such as cyclophosphamide or azathioprine (9, 11–14). However, sustained disease control remains difficult in some refractory patients. In the present case, the patient received multiple immunosuppressive regimens during the course of the disease but still developed glucocorticoid dependence, repeated relapse during tapering, and substantial treatment-related adverse effects, thereby further highlighting the limitations of conventional immunosuppressive therapy.

Rituximab has been recommended as a first-line therapeutic option in AAV, but its role in ANCA-associated HP remains unclear. A small number of case reports and limited case series have suggested that rituximab may reduce dural inflammation and improve clinical symptoms by targeting B cells and reducing ANCA production (15–17). In previously reported cases of ANCA-associated hypertrophic pachymeningitis, rituximab has generally been administered according to standard AAV regimens, such as 375 mg/m^2^ weekly for 4 weeks or 1 g given twice 2 weeks apart (16–18), with maintenance doses usually ranging from 500 to 1,000 mg every 4–6 months. Whether fixed intervals and standard doses are necessary in all cases remains uncertain.

In our patient, no systemic organ involvement was evident initially, but the disease was characterized by frequent relapse, intolerance to multiple immunosuppressive agents, and an increased risk of infection during long-term treatment. In this context, particularly for patients with relapsing or refractory disease, we explored whether an even lower-dose rituximab regimen could sustain B-cell depletion and reduce relapse risk, while mitigating clinical concerns associated with conventional regimens, including infection risk, infusion burden, and treatment costs. Referring to the individualized re-dosing strategy used in the MAINRITSAN 2 trial (15, 19), and guided by CD19^+^ B-cell counts and ANCA titers, rituximab was administered as an individualized, on-demand maintenance regimen at 100 mg per infusion, which was substantially lower than conventional induction and maintenance regimens.

Such an ultra-low-dose rituximab regimen has previously been reported in ANCA-associated nephritis, with no substantial difference in efficacy compared with cyclophosphamide and with a favorable safety profile (20). In other autoimmune diseases, including systemic lupus erythematosus (SLE)-associated autoimmune hemolytic anemia, immune thrombocytopenia (ITP), and mixed connective tissue disease (CTD), studies have shown that rituximab administered at 100 mg once weekly for 4 consecutive weeks has comparable efficacy to the standard regimen of 375 mg/m^2^ weekly for 4 weeks, although B-cell reconstitution occurs earlier with the low-dose regimen (21–24). These findings suggest that individualized low-dose treatment regimens can be attempted.

An additional noteworthy finding in this case was the dissociation between clinical response and serological or inflammatory indices. Although low-dose rituximab led to marked improvement in symptoms and radiological findings, cerebrospinal fluid pressure, MPO-ANCA titers, and inflammatory markers such as ESR and CRP did not decline in parallel (Figures 2B,C). Persistent MPO-ANCA positivity after rituximab treatment can also be partly explained by the pharmacological mechanism of rituximab. First, studies have shown that, because rituximab has a relatively large molecular weight of approximately 145 kDa, when the blood–brain barrier is intact, rituximab concentrations in the CSF are only 0.1–4.4% of serum concentrations, resulting in insufficient rituximab penetration across the blood–brain barrier. Second, rituximab primarily depletes CD20-positive B cells, whereas terminally differentiated antibody-producing cells, particularly long-lived plasma cells, do not express CD20 and therefore are not directly eliminated by rituximab (25, 26). In the present case, only peripheral blood B-lymphocyte counts were monitored to evaluate treatment response. CD19^+^ B lymphocytes in the CSF were not serially assessed, which may have led to underestimation of ongoing intrathecal immune activity. Once long-lived plasma cells become established in the bone marrow or secondary lymphoid organs, they may continue to produce ANCA despite B-cell depletion (25, 27).

In addition, CRP may serve as a potential adjunctive biomarker for assessing treatment response in ANCA-associated HP. In a case series of rituximab-treated ANCA-associated HP, CRP levels decreased after treatment in all patients and paralleled clinical and radiological improvement (16). In another cohort of AAV patients, follow-up MRI was performed when worsening headache or cranial neuropathy was accompanied by elevated CRP, suggesting that CRP may be useful as a practical inflammatory indicator during clinical monitoring (28). Previous reports have also described elevated ESR and CRP as common laboratory abnormalities in MPO-ANCA-associated HP (29). Consistent with these observations, CRP showed a similar decreasing trend after intensified immunosuppressive treatment in the present patient (Figure 2B). Taken together, these limited data suggest that CRP, and possibly ESR, may serve as adjunctive markers for assessing treatment response, although they should be interpreted together with clinical symptoms, MRI findings, CSF parameters, and ANCA titers rather than used as standalone indicators.

Therefore, in this case, the improvement in symptoms and MRI findings may reflect control of local dural inflammation, whereas the persistent MPO-ANCA positivity and elevated inflammatory markers suggest residual systemic immune activity. The patient’s prior intolerance to multiple immunosuppressive agents and irregular treatment may also have affected the continuity of immunosuppressive therapy (30). In this context, inflammatory markers, MPO-ANCA levels, and MRI findings may still serve as useful reference indicators for monitoring disease activity, although they may not reliably predict relapse on an individual basis (16, 19, 31).

### Limitations

Several diagnostic limitations should also be acknowledged. First, contrast-enhanced magnetic resonance imaging (MRI) is the main imaging modality for evaluating hypertrophic pachymeningitis and can demonstrate focal or diffuse dural thickening and enhancement (32). These characteristic MRI findings provide important diagnostic clues. They are also useful for assessing disease extent and monitoring treatment response. However, they are not disease-specific and cannot independently determine the underlying etiology. Dural biopsy provides the most definitive histopathological evidence for the diagnosis of hypertrophic pachymeningitis and is important for excluding alternative etiologies such as malignancy, infection, and IgG4-related disease. However, in this case, dural biopsy was not performed because the patient refused this invasive procedure due to its potential procedural risks. Therefore, the diagnosis of ANCA-associated hypertrophic pachymeningitis was not histopathologically confirmed. Second, although the available clinical findings did not suggest spinal cord or nerve root involvement, spinal MRI may be useful for evaluating possible spinal meningeal, spinal cord, or nerve root involvement in patients with hypertrophic pachymeningitis. Therefore, the absence of spinal MRI limited the assessment of the full extent of meningeal disease. Third, whole-body CT or 18F-FDG PET-CT was not performed. These examinations may help identify occult malignancy, infection, or systemic inflammatory lesions and may provide guidance for biopsy site selection (33, 34). Although the available cerebrospinal fluid, laboratory, and chest imaging findings did not support malignancy or infection in this case, whole-body imaging could have made the exclusion of these alternative diagnoses more robust.

## Conclusion

In summary, this case suggests that MPO-ANCA-associated HP may belong to the spectrum of AAV and that systemic involvement may emerge only after a prolonged disease course, underscoring the need for long-term follow-up and dynamic reassessment. For patients with relapsing or refractory disease, particularly those who are intolerant to immunosuppressive agents or at high risk of infection, an individualized low-dose rituximab regimen may represent a potential therapeutic option. However, because this is a single-case observation, the level of evidence remains limited, and the optimal dose, dosing interval, and target population for rituximab in this setting require further investigation.

## Data Availability

The original contributions presented in the study are included in the article/supplementary material, further inquiries can be directed to the corresponding author.
